# Students at the Centre: Establishing and Delivering a National Student Psychiatry Conference at a New UK Medical School

**DOI:** 10.1192/bjo.2026.11285

**Published:** 2026-06-30

**Authors:** Sophie Krug, Keiru Ajuzieogu, Ella Haines, Priscilla Yowa, Joanne Rodda, Ramneek Kaur, Naomi King, Doreen Kuek, Alexander Lawrence, Jamie Mawer, Swara Rajurkar, Sanem Ucur, Leera Vite, Joseph Kendall, Sherlie Arulanandam, Subodh Dave, Declan Hyland, Chris Holland, Sukhi Shergill

**Affiliations:** 1 Kent and Medway Medical School, Canterbury, United Kingdom; 2 Kent and Medway Mental Health NHS Trust, Maidstone, United Kingdom

## Abstract

**Aims:**

Kent and Medway Medical School (KMMS), a new UK medical school, hosted the 2026 National Student Psychiatry Conference. The conference was organised and delivered by a student committee supported by faculty and the RCPsych *Choose Psychiatry* team. We describe the learning around the planning and delivery of the conference, and consider its wider educational and institutional impact.

**Methods:**

A student organising committee was established with defined roles and responsibilities, supported by frequent planning meetings and oversight by faculty and the *Choose Psychiatry* team. A conference theme and associated social activities were agreed.

**Results:**

Twelve KMMS students from different year groups led the planning and delivery of the conference; roles included programme development, speaker liaison, sponsorship/budgeting, communications, accommodation, and logistics. Clear role allocation and regular meetings supported coordination of a complex project alongside academic commitments. The weekend conference received overwhelmingly positive feedback regarding organisation, atmosphere and session quality from the 103 delegates and 21 speakers (98% overall satisfaction).

Committee members described developing advanced professional communication, project management and leadership skills under time and resource constraints. Learning extended beyond individual task completion to coordinating interdependent workstreams, negotiating priorities, and responding collectively to challenges. They also described gaining practical experience of leadership within a complex system, including budget management, sponsorship planning, using organisational tools (e.g. spreadsheets, booking systems), and real-time problem solving to manage technical and timetabling issues.

Reflecting on this, students identified improvements for future events, including starting external communications earlier, longer breaks between sessions, ordering merchandise further in advance, and greater delegation on the day.

Despite the workload, students found the experience rewarding. Working as a team towards a shared goal, seeing plans come together, and positive feedback generated a strong sense of enjoyment and achievement, alongside practical learning opportunities rarely available within undergraduate medical education.

**Conclusion:**

Medical students can develop meaningful leadership capability when trusted to lead complex, collective projects with appropriate support. Beyond promoting psychiatry, the conference provided experiential learning in systems-based working and medical leadership. For the medical school and RCPsych, the conference raised the profile of psychiatry within undergraduate training and strengthened national connections. Investing in student-led conferences provides a practical way to support engagement with psychiatry and contribute to long-term recruitment and workforce development.

